# Tailoring optical and ferroelectric properties in Sb_1−*x*_Bi_*x*_SI van der Waals chalcohalides towards solar absorber applications

**DOI:** 10.1039/d5ta07038d

**Published:** 2025-11-27

**Authors:** Sara A. López-Paz, Harish K. Singh, Alba S. J. Méndez, Volodymyr Multian, Jeremie Teyssier, Ulrich Aschauer, Fabian O. von Rohr

**Affiliations:** a Department of Chemistry, University of Copenhagen Universitetsparken 5 DK-2100 Copenhagen Denmark salp@chem.ku.dk; b Department of Chemistry and Physics of Materials, University of Salzburg 5020 Salzburg Austria; c Deutsches Elektronen-Synchrotron (DESY) Notkestraße 85 22607 Hamburg Germany; d Department of Quantum Matter Physics, University of Geneva 24 Quai Ernest-Ansermet Geneva CH-1211 Switzerland fabian.vonrohr@unige.ch

## Abstract

The efficiency of solar cells depends on absorber materials whose optical and electronic properties can be precisely tuned for optimal performance. The mixed-anion, van der Waals material SbSI is a promising candidate, offering both a narrow bandgap and strong ferroelectric polarization, which together produce a robust bulk photovoltaic effect and high power conversion efficiency (PCE). Here, we demonstrate that substituting bismuth for antimony in SbSI enables controlled tuning of both the bandgap and ferroelectric properties. Increasing Bi content induces anisotropic changes in the crystal structure, with the bandgap decreasing rapidly at low substitution levels and stabilizing at 1.5 eV for higher Bi concentrations. Concurrently, ferroelectricity is strongly suppressed, as evidenced by the disappearance of soft-phonon modes and second-harmonic generation signals. First-principles calculations support the observation that the ferroelectric ground state becomes unfavorable with Bi substitution, driven by changes in Sb/Bi–S bonding characteristics and a concomitant reduction in the lone-pair expression. This ability to finely tune both structural and functional properties highlights the potential of Sb_1−*x*_Bi_*x*_SI for the development of high-performance, next-generation solar-energy materials.

## Abstract 2

The efficiency of solar cells depends on absorber materials with tunable optical and electronic properties. Here we explore the effect of Sb by Bi substitution on the optical and ferroelectric properties of Sb_1−*x*_Bi_*x*_SI chalcohalides.

## Graphical abstract

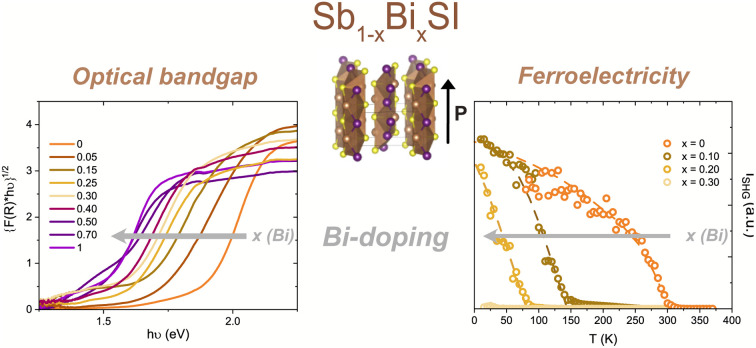

## Introduction

Metal halides have emerged as a versatile family of optoelectronic materials for energy applications. In particular, *n*s^2^-based metal halides are being intensively explored as solar absorbers, photocatalysts and emissive materials.^1^ Perovskite halides AMX_3_, combining covalent MX_3_ (M = Sn, Pb; X = I, Br, Cl) networks and organic/inorganic A units (A^+^ = Cs^+^, Rb^+^, CH_3_NH^+^_3_, NH_2_(CH)NH^+^_2_, *etc.*) are one of the most promising families. With bandgaps in the 1.10–1.55 eV range and high carrier mobilities arising from *n*s(M)–2p(X) hybridization, in combination with high dielectric screening from the hosted organic units, these materials achieve high power conversion efficiency (PCE) in solar-cell devices.^2–4^ The possibility of reducing the dimensionality expands the myriad of functionalities, providing space for the architecture of organic–inorganic networks with different connectivities.^5^

The role of the *n*s^2^ lone pair in the optical properties of perovskite halides containing Sn^2+^, Pb^2+^, or Bi^3+^ has been the focus of intense consideration.^6–9^ Lone pairs are known to impose structural constraints due to their stereochemical activity, with a tendency to off-center to accommodate a lobe-like asymmetric electron density. Their role in promoting structural distortions and in this way driving ferroic behaviours has been demonstrated in a wide range of ferroic materials, including BiFeO_3_, PbTiO_3_ or BiMnO_3_.^10^ While classically overlooked, the active role of the anions as accounted for in the revised lone-pair model^11–13^ for lone pair activity is getting increasing recognition. The presence of antibonding states at the valence band maximum (VBM), arising from *n*s^2^–X*n*p hybridization, has been highlighted as a key factor to optimize optical absorption in halide perovskites, by lifting the VBM and in providing defect tolerance.^2,3,6^ This has also been proven to be behind the exceptional photocatalytic activity and stability of Sillen–Aurivillius type oxyhalides for visible light-induced water splitting.^14–16^ Similarly, their impact on the thermoelectric and dielectric properties has also been discussed (*e.g.*, in MnSb_2_O_4_, CuSbSe_2_, and CuBiSCl_2_).^17,18^

In this context, van der Waals bismuth and antimony-based chalco-halides stand as an exceptional system to evaluate the effect of the lone pair expression on the optical properties. These material classes have recently garnered attention as potential solar absorbers,^19–21^ due to their suitable bandgaps (1.3–3 eV) and exceptional carrier mobilities. While still having lower figures of merit than halide perovskites, the outstanding performance of SbSI, SbSeI, BiSI and BiSeI as photovoltaic materials has been highlighted.^22–25^ This, combined with their low toxicity and easy processability,^26^ raises the potential of chalcohalides to enable next-generation photovoltaic devices.^27,28^

Among these materials, SbSI is of the greatest interest as a prototypical ferroelectric material, developing high polarization values below the *T*_c_ located at room temperature.^29^ The combination of ferroelectricity and solar absorption in SbSI drives a robust bulk photovoltaic effect^30,31^ of both fundamental interest and technological potential. The occurrence of long-lived shift photocurrents and a complex coupling of charge and lattice dynamics have been further demonstrated in SbSI.^32–34^ Partial replacement of Sb by Bi has been shown to further increase the PCE,^35^ presumably due to the decrease in the bandgap towards the optimal solar absorption range and thus providing the possibility of band alignment in ferroelectric–paraelectric heterostructures.^36,37^ The earliest reports on the effect of Bi suggest a depression of the ferroelectric properties based on dielectric measurements, but controversy between reports highlights a challenge in chemical homogeneity.^38–40^ We revisit this question here by synthesizing a complete solid solution by chemical vapour transport (CVT).

We consider Sb_1−*x*_Bi_*x*_SI as a model system to study the interplay between lone pair expression, optical absorption, and ferroelectric properties. We address here the evolution of the structural, electronic, and ferroelectric properties across the Sb_1−*x*_Bi_*x*_SI solid solution by combining temperature-dependent synchrotron X-ray diffraction, UV-Vis spectroscopy, and combined Raman and second harmonic generation spectroscopy measurements. This systematic study, in conjunction with DFT calculations, highlights the strong impact of the lone-pair expression on the structural, electronic, and ferroic behavior that follows the substitution of Sb by Bi in SbSI. We highlight that a compromise between the effect of Bi substitution on the optical bandgap and the ferroelectric properties has to be considered when designing solar cells based on SbSI–BiSI heterojunctions.

## Experimental methods

### Crystal growth

Sb_1−*x*_Bi_*x*_SI (*x* = 0–1) single crystals were grown by CVT using elemental bismuth (Merck, 99.99%), antimony (Thermo Scientific, 99.9999%), sulfur (ACROS ORGANICS, 99.999%), and iodine (Fluka, >99.8%). Stoichiometric amounts of starting materials were ground, mixed and sealed under vacuum in a quartz ampule (12 cm in outer diameter and 10–12 cm long) and subjected to thermal treatment in a temperature gradient of 550–500 °C. As-grown SbSI crystals are red and have a needle morphology, while Bi-containing crystals become more brittle and darker with increasing Bi content. The samples are air-stable. Initial characterization was performed by powder X-ray diffraction (PXRD) using a Rigaku SmartLab diffractometer. Compositional homogeneity was checked by energy-dispersive X-ray spectroscopy (EDS) using a JEOL JSM-IT800 scanning electron microscope.

### Synchrotron X-ray diffraction (XRD) experiments

Temperature-dependent synchrotron XRD measurements were performed at the P02.1 Powder Diffraction and Total Scattering beamline at PETRA III-DESY (Hamburg, Germany). Measurements were performed with a wavelength of *λ* = 0.2073 Å using a Varex XRD 4343CT flat panel detector (150 × 150 µm^2^ pixel size; 2880 × 2880 pixel area) with a sample-to-detector distance (SDD) of 1021 mm. Calibration of the detector and the SDD was performed using LaB_6_ (NIST 660b) as the standard material. The powdered samples were packed in capillaries of 0.3 mm diameter and measured in the temperature range of 100–300 K using a cryostream under spinning. Data analysis by the Rietveld method was performed using the FULLPROF SUITE package.^41^

### Diffuse reflectance UV-Vis spectroscopy

Diffuse reflectance spectroscopy measurements were performed in a Shimadzu UV-2600 spectrophotometer using an integrating sphere. The powder samples were deposited on top of BaSO_4_ and measured at room temperature in the range of 400–1400 nm. Measurements were recorded in reflectance mode and Tauc plots were calculated by using the Kubelka–Munk function for determination of the electronic band gap. See SI Note 1 for further details.

### Raman and second harmonic generation measurements

Raman and Second Harmonic Generation (SHG) measurements were performed on a unique instrument that couples a Ti:sapphire laser (Coherent Vitesse 800 nm, 100 fs, 80 MHz) to a commercial Raman spectrometer (Horiba LabRAM HR Evolution) using a patented optical module^42^ allowing simultaneous recording of polarization resolved Raman, PL and SHG signals. The samples are glued (silver paint) on the cold finger of a He flow cryostat (Konti Micro from CryoVac GmbH) and the Raman spectra are acquired in the confocal configuration with a spectral resolution of 0.3 cm^−1^ (0.04 meV). Laser beams from a Raman continuous wave laser emitting at 532 nm and ultrafast laser at 800 nm are focused on the same spot with sizes ranging from 0.4 to 1.0 µm (FWHM). Raman signal acquisition was performed with an LN cooled Si charge coupled device (CCD) array (Horiba Scientific Symphony II). The Raman laser power is set to 200 µW and the temperature is monitored using the Raman Stokes/anti-Stokes ratio as implemented in the Speqqle program. The SHG signal at 400 nm is collected in epidetection geometry in a separate optical channel using a LN-cooled CCD array (Princeton Instruments). A cascade of 3 band-pass interference filters rejects the excitation laser light (ThorLabs FBH400-10, CWL = 400 nm, FWHM = 10 nm). The average power of the pump beam is set to 10 mW which corresponds to a peak power density of 150 GW cm^−2^. The laser heating is again estimated by Raman-based thermometry performed from the same spot.

### Computational details

Density functional theory (DFT) calculations were performed using the projector augmented wave (PAW) method^43,44^ as implemented in the VASP code.^45–48^ The exchange–correlation energy was treated with the screened hybrid functional HSE06,^49,50^ and we used 25% exact Hartree–Fock exchange together with the Perdew–Burke–Ernzerhof (PBE) generalized-gradient approximation.^51^ A plane-wave energy cutoff of 520 eV and Gaussian smearing with a broadening width of 0.01 eV were employed for all calculations. Long-range van der Waals interactions were included using Grimme's DFT-D3 dispersion correction.^52^ Integration of the Brillouin zone was performed using a Monkhorst–Pack^53^ 4 × 3 × 8 *k*-point mesh for structural optimization and a 4 × 5 × 6 mesh for phonon calculations. Geometry optimizations were performed until the residual force on each atom was less than 0.001 eV Å^−1^. GGA-PBE fails to reproduce the ferroelectric state: the polar phase relaxes to identical *c*-coordinates for Sb, S, and I, becoming non-polar.^31^ Moreover, the GGA-PBE phonon spectrum of the centrosymmetric *Pnam* (no. 62) phase is free of imaginary modes, whereas at least one soft mode (saddle-point instability) is expected to drive the transition to the polar *Pna*2_1_ (no. 33) phase. In contrast, HSE (25% exact exchange) recovers this instability and yields internal coordinates in better agreement with the experiment. The calculated lattice parameters agree well with the experimental values, showing a deviation of less than 1%, except for the *b* lattice parameter of BiSI, which is 1.785% smaller than the experimental value (Table S4). This does not affect the electronic properties, as verified by comparing the DOS with that obtained using the experimental lattice parameters (Fig. S15). We generally used relaxed lattice parameters, only performing charge density calculations for SbSI in the *Pna*2_1_ phase using the experimental lattice parameters, to benchmark our results with the study of Amoroso *et al.*^31^

Phonon dispersions were computed within the harmonic approximation using the frozen phonon method and analyzed with the phonopy package.^54^ A 1 × 1 × 3 supercell was employed to construct the force constant matrix, based on forces evaluated at the HSE06 level of theory. Chemical bonding analysis was performed using the crystal orbital Hamilton population (COHP) method^55,56^ as implemented in the LOBSTER code.^57^ Post-processing electronic structure analysis was performed using the VASPKIT package.^58^

## Results and discussion

### Crystal structure evolution across the Sb_1−*x*_Bi_*x*_SI solid solution

**Fig. 1:**
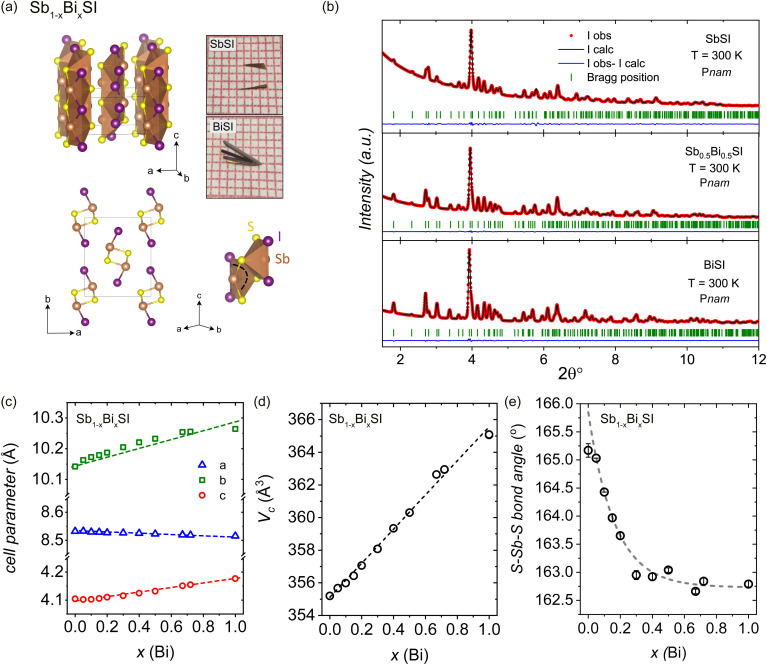
(a) Crystal structure of Sb_1−*x*_Bi_*x*_SI along the two main crystallographic directions, showing the coordination polyhedra around the Sb/Bi cations. Optical microscope image of as-grown single crystals for SbSI and BiSI. (b) Rietveld refinement of the RT SXRD patterns for Sb_1−*x*_Bi_*x*_SI (*x* = 0, 0.5, and 1) within the *Pnam* space group. (c–e) Variation of the cell parameters, unit cell volume (*V*_c_), and S–Sb–I bond angle as a function of the Bi content in Sb_1−*x*_Bi_*x*_SI as derived from Rietveld refinement of the synchrotron powder X-ray diffraction patterns at room temperature.

To understand how Bi substitution affects the structure and properties of SbSI, we first characterized the pristine material's crystal structure at room temperature. The room temperature synchrotron XRD pattern for SbSI was refined within the *Pnam* space group, and the refined cell parameters agree with the previously reported orthorhombic structure with cell dimensions *a* = 8.5326(4) Å, *b* = 10.1406(5) Å, and *c* = 4.1042(2) Å (compare ref. 59). The structural model is shown in Table 1 and the refined crystal structure is shown in Fig. 1a. Sb cations show square pyramidal coordination, with two iodine and two sulfur atoms occupying the basal anionic positions. The pyramids share edges along the *a*-axis, resulting in double SbSI chains running along the *c*-axis. Notably, the double chains interact *via* weak van-der-Waals forces along the *ab* plane, conferring a pronounced one-dimensional character. This is a consequence of the stereo-chemical activity of the lone pair leading to asymmetric coordination, with an off center displacement of the Sb cations in the square pyramidal [SbS_3_I_2_] units, with an S–Sb–I angle of approximately 165°.

**Table 1:** Refined structural parameters for SbSI at 300 K and 100 K within the *Pnam* and *Pna*2_1_ space groups, respectively, as derived from the Rietveld refinement of the synchrotron XRD data

Atom	Site	*x*	*y*	*z*	Biso	Occ.
***T* = 300 K *Pnam*** a						
Sb	4c	0.1199(2)	0.1232(2)	0.25	1.1(1)	1
S	4c	0.8445(6)	0.0478(5)	0.25	1.4(1)	1
I	4c	0.5074(2)	0.8276(1)	0.25	1.73(5)	1

***T* = 100 K *Pna*2**_**1**_ b						
Sb	4a	0.1199(1)	0.1235(1)	0.3141(5)	0.39(4)	1
S	4a	0.8442(5)	0.0490(4)	0.274(2)	0.65(9)	1
I	4a	0.5075(2)	0.8279(1)	0.25	0.31(4)	1

a *a* = 8.5326(4) Å; *b* = 10.1406(5) Å; *c* = 4.1042(2) Å: *V*_c_ = 355.13(3) Å^3^; *R*_Bragg_ = 2.36; *R*_p_ = 0.647; *R*_wp_ = 1.02.

b *a* = 8.4959(3) Å; *b* = 10.0763(4) Å; *c* = 4.1222(2) Å: *V*_c_ = 352.90(2) Å^3^; *R*_Bragg_ = 1.98; *R*_p_ = 0.596; *R*_wp_ = 0.935.

With decreasing temperature, SbSI is known to undergo a displacive ferroelectric transition, characterized by cooperative shifts of S and Sb ions along the *c*-axis.^59^ The use of the *Pnam* space group assumes a paraelectric state, with cations at the special 4c positions (see Table 1). In the ferroelectric state, the ionic displacements along the polar *c*-axis are captured by freeing the *z* coordinate, resulting in a reduction of symmetry to a *Pna*2_1_ space group. As the ferroelectric transition of SbSI is located close to RT, refinement of the RT SXRD data for SbSI was conducted using both structural models, as shown in Fig. S1 and Table S1. Although slightly improved agreement factors are obtained when using the *Pna*2_1_ space group, the refined *z* coordinates reflect minor atomic displacements, and the refined cell parameters using both structural models show no significant variation. This suggests that, at room temperature, the structural signature of ferroelectricity is subtle and challenging to resolve with synchrotron XRD alone. For consistency, all refinements for Sb_1−*x*_Bi_*x*_SI at room temperature were conducted within the *Pnam* space group, assuming a paraelectric state.

The Sb_1−*x*_Bi_*x*_SI solid solution retains the parent structure type across the full composition range, with a progressive shift of the reflections towards higher angles (see Fig. S2), associated with the overall expansion of the unit-cell volume, as expected from substitution of Sb by Bi due to the increased ionic radius of the 6s cation. To characterize the unit cell evolution across the solid solution, we performed Rietveld refinement of the RT synchrotron XRD patterns within the *Pnam* space group for Sb_1−*x*_Bi_*x*_SI (*x* = 0–1). Fig. 1b shows the Rietveld refinement of the *x* = 0, 0.5, and 1 members, using the same structural model within the *Pnam* space group. The refined cell parameters and unit cell volume (*V*_c_) are shown in Fig. 1c and d as a function of *x*(Bi), highlighting the anisotropic variation along the three main crystal axes. The refined cell parameters are listed in Table S3.

Overall, the unit-cell volume increases across the whole compositional range. On the other hand, a contraction of the *a* axis is observed upon Bi substitution, reflecting the tendency of Bi atoms to increase their coordination number, with a shortening of the next neighbor Sb/Bi–I distances along the *a* axis. This anisotropic contraction of the unit cell reflects the structural constraints of the *n*s^2^ lone pair, which is directed along the *a*-axis (see partial density maps in Fig. 6). The Bi incorporation also results in a further displacement of the Sb/Bi cations from the center of the square pyramidal units. As shown in Fig. 1e, the S–Sb–I angle shows a marked decrease, reaching saturation for *x*(Bi) > 0.5.

### Electronic band-gap evolution

Tuning the electronic bandgap is critical for optimizing solar absorber materials, as it directly influences light absorption and device efficiency. Pristine SbSI exhibits an indirect bandgap of 1.8–2.1 eV, whereas BiSI has a narrower bandgap of 1.5 eV.^19,35,60^ In the Sb_1−*x*_Bi_*x*_SI solid solution, a linear interpolation of bandgap values might be expected according to Vegard's law; however, the pronounced anisotropic structural changes observed with Bi incorporation may anticipate a more complex behavior, with a non-linear bandgap evolution.^61^

**Fig. 2:**
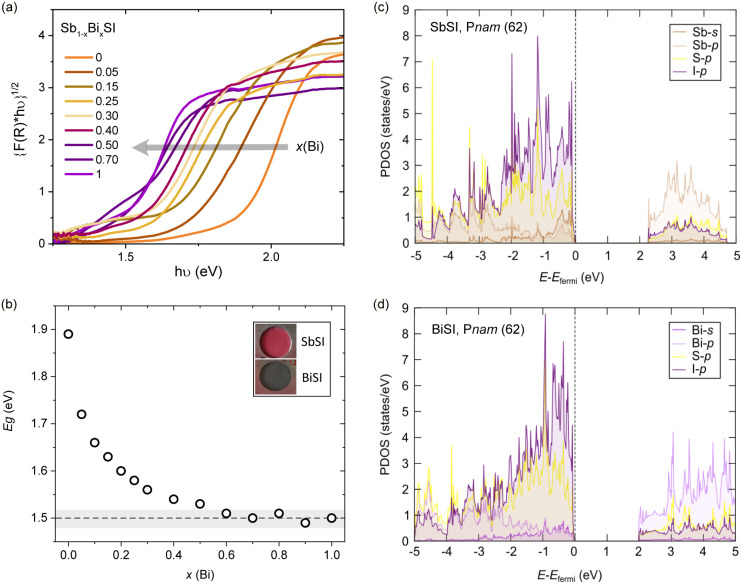
Optical properties of Sb_1−*x*_Bi_*x*_SI. (a) Tauc plot assuming an indirect bandgap for Sb_1−*x*_Bi_*x*_SI, showing a progressive red shift with *x*(Bi), and (b) the extracted bandgap values as a function of *x*(Bi). Projected densities of states including spin–orbit coupling for (c) SbSI and (d) BiSI in the *Pnam* space group.

To characterize the bandgap variation across the solid solution, we performed diffuse reflectance spectroscopy measurements. The Tauc plot is shown in Fig. 2a. Our analysis assumes an indirect bandgap for Sb_1−*x*_Bi_*x*_SI mixed compositions (with *γ* = 2, see SI Note 1 for details), as observed for the two end members SbSI and BiSI. While the bandgap evolution derived from this analysis is qualitatively reproduced when considering direct transitions, the possibility of indirect to direct bandgap renormalization in mixed SbSI–BiSI crystals may deserve further analysis, as both SbSI and BiSI show close direct transitions.^24,31^ A progressive shift of the reflectance edge towards lower photon energies is observed with increasing Bi substitution, reflecting the decrease in the bandgap values from 1.9 eV in SbSI to 1.5 eV in BiSI. For the estimation of the bandgap, the intersection of the extrapolation of the linear part of the Tauc plot with the photon energy is considered (see Fig. S3).^62^ The obtained bandgap values as a function of composition are shown in Fig. 2b. A non-linear behaviour is observed, with a strong depression of the bandgap for very low substitution levels. The fast decrease for *x*(Bi) < 0.5 is followed by saturation above *x*(Bi) ∼ 0.5 at bandgap values of ∼1.5 eV, falling in the optimal bandgap region for solar absorption. Further analysis of the effect of Bi substitution on the absorption coefficient and current densities will be required to evaluate the overall impact on solar absorption efficiency, as previously noted for Sb_1−*x*_Bi_*x*_SI.^35^

Our DFT calculations for the refined structures at room temperature qualitatively reproduce the reduction of the bandgap for the two end members. The computed partial density of states (Fig. 2c and d) shows the valence band to be composed primarily of broad Sb/Bi-5p/6p, I-5p, and S-3p hybridized states, in qualitative agreement with previous calculations.^31^ However, a significant contribution of Bi-6s and Sb-5s states is also observed at the VBM. As discussed below, based on COPH calculations, this reflects the presence of antibonding states at the top of the valence band, with crucial implications for the lone pair expression. On the other hand, the conduction band is dominated by Bi-6p and I-5p/S-3p orbitals. For SbSI, the band gap decreases from 2.47 eV (without SOC) to 2.27 eV (with SOC); for BiSI, it drops from 2.55 eV (without SOC) to 2.01 eV (with SOC). Bi exhibits stronger spin–orbit coupling than Sb, which results in a broader conduction-band tail in BiSI due to enhanced p–p hybridization (Fig. 2c and d). Consequently, SOC reduces the band gap more in BiSI (0.54 eV) than in SbSI (0.20 eV). To provide a more accurate comparison with experiments, we also used a reduced fraction of exact exchange of 10% in HSE, obtaining a band gap of 1.88 eV for SbSI and 1.61 eV for BiSI, consistent with reported optical measurements.

A similar bandgap reduction has been observed in SbSI above a critical pressure of ≈15 GPa.^63^ The reduction of the *a*-axis observed here with the incorporation of Bi resembles the 1D to 3D crossover in SbSI under pressure, with the formation of additional Sb–I bonds. However, while the overall unit-cell contraction in SbSI under pressure induces a PE to FE distortion at room temperature, Bi incorporation suppresses ferroelectricity, as we will discuss below.

### Ferroelectric distortion in Sb_1−*x*_Bi_*x*_SI

We now examine the evolution of the crystal structure in the ferroelectric state. Early studies reported that Bi doping leads to a pronounced decrease in the ferroelectric transition temperature (*T*_c_), although subsequent measurements of the dielectric function yielded inconsistent results.^38,39^ Pristine SbSI undergoes a displacive ferroelectric transition, characterized by cooperative shifts of S and Sb ions along the *c*-axis. This symmetry-lowering distortion transforms the structure from centrosymmetric *Pnam* into polar *Pna*2_1_, resulting in a spontaneous polarization along the *c*-axis.

A striking feature of many ferroelectric materials—including SbSI—is the occurrence of negative thermal expansion (NTE) along the polar axis in the ferroelectric phase. This phenomenon is thought to arise from strong coupling between lattice distortions and the development of spontaneous polarization, although the underlying mechanisms can be complex and material-specific.^64–66^ NTE is not unique to classical ferroelectrics such as BaTiO_3_ or PbTiO_3_, but also appears in a range of van der Waals materials and strongly correlated systems, including CrSBr,^67^ LiCrTe_2_,^68^ CrBr_3_,^69^ and even graphene,^70^ where lattice, electronic, and magnetic orders are intricately linked. We observe clear signatures of negative thermal expansion in several members of the Sb_1−*x*_Bi_*x*_SI series, further underscoring the interplay between lattice dynamics and electronic order in these low-dimensional materials.

**Fig. 3:**
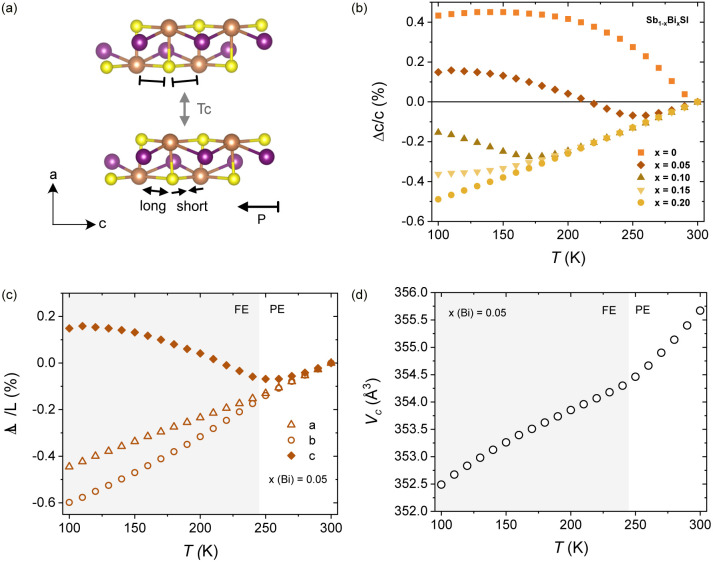
Temperature dependent structural evolution in Sb_1−*x*_Bi_*x*_SI. (a) Crystal structure of SbSI showing the distortion involved in the symmetry lowering below *T*_c_, resulting in non-equivalent Sb–S bonds along the *c*-axis. (b) Temperature dependence of the *c*-axis for Sb_1−*x*_Bi_*x*_SI (*x* = 0–0.3) showing the uniaxial negative thermal expansion of the polar axis below *T*_c_. The temperature dependence of the three unit cell parameters and the cell volume for *x* = 0.05 is shown in (c) and (d), with a change in the slope at *T*_c_.

The refinement of the 100 K SXRD data for SbSI was conducted using the reported *Pna*2_1_ model for the ferroelectric state (Table 1).^59^ Details of the fitting are shown in Fig. S4 and Table S2. A good agreement is obtained with this model, with a substantial displacement of the Sb and S ions along the *c*-axis, as reflected in the shift of the *z*-coordinate, no longer at a special position. We have confirmed that fitting with the *Pnam* model results in worse agreement factors. In particular, the *Pnam* model fails to reproduce the relative intensity of the (210) and (011) reflections (see Fig. S4). The shift of Sb and S leaves one long and one short Sb–S distance, as sketched in Fig. 3a. This distortion relaxes progressively with increasing temperature, converging to an average Sb–S distance at the *T*_c_ (see Fig. S5). The structural distortion is further reflected in a change in relative intensity for the (210) and (011) reflections, as shown in Fig. S6. It is, however, difficult to assign a precise *T*_c_ for this broad, first-order transition. Therefore, in order to capture the structural distortion, the temperature evolution of the cell parameters was refined using the *Pna*2_1_ model.

The temperature evolution of the *c*-axis for different compositions is shown in Fig. 3b, and the variation of the cell parameters and unit cell volume for *x* = 0.05 is shown in Fig. 3c and d. A clear change in the slope of the unit cell volume is observed with decreasing temperature, followed by a pronounced negative thermal expansion of the *c*-axis, signaling the structural distortion at the paraelectric (PE) to ferroelectric (FE) transition. When comparing different compositions in Sb_1−*x*_Bi_*x*_SI, a decrease in transition temperature with increasing Bi content is observed, as evidenced in Fig. 3b. No structural transition is observed for *x* > 0.20 in the measured temperature range (100–300 K). It remains a question whether the depression of the T_c_ and the significant reduction of the thermal expansion coefficient (see Fig. S7) is followed by a concomitant decrease in polarization values, which may deserve further studies.

**Fig. 4:**
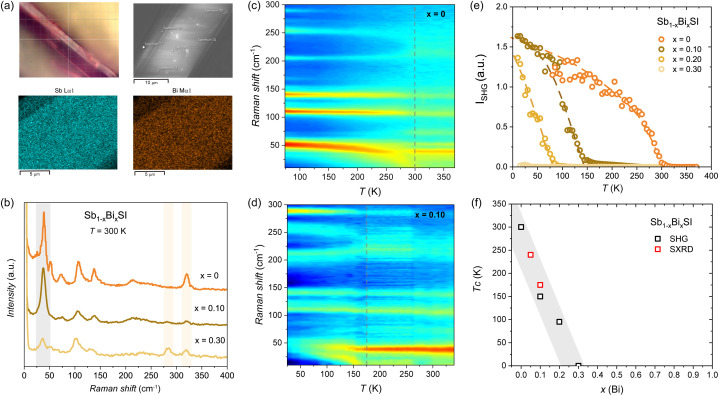
Raman and SHG measurements for Sb_1−*x*_Bi_*x*_SI. (a) Micrograph of the measured area and the corresponding SEM image and EDS maps (see Fig. S8–S10). (b) Unpolarized Raman spectrum at 300 K for selected compositions and temperature color plots for (c) *x* = 0 and (d) *x* = 0.10 showing the softening of the phonon mode below 50 cm^−1^ at *T*_c_. (e) SHG intensity for Sb_1−*x*_Bi_*x*_SI (*x* = 0, 0.10, 0,2 and 0.30). Dotted lines are a guide to the eye. (f) Variation of the *T*_c_ as a function of *x*(Bi) as determined from the SHG and SXRD.

In order to verify the local *vs.* average structural distortion, we performed Raman experiments. Raman measurements were performed using a micro-focused beam, probing an area of ∼1 µm. We have performed EDS on the probed areas, ensuring compositional homogeneity (see Fig. 4a and S8–S10). Raman spectra at room temperature of *x* = 0, 0.1, 0.3 compositions are shown in Fig. 4b. The observed active Raman modes for SbSI (*x* = 0) at room temperature are in good agreement with previous reports in the paraelectric state.^71^ The incorporation of Bi is reflected in a two-mode behaviour of the higher energy mode, with an additional peak observed at 280 cm^−1^ (see the red frame in Fig. 4b), as previously reported.^38^

**Fig. 5:**
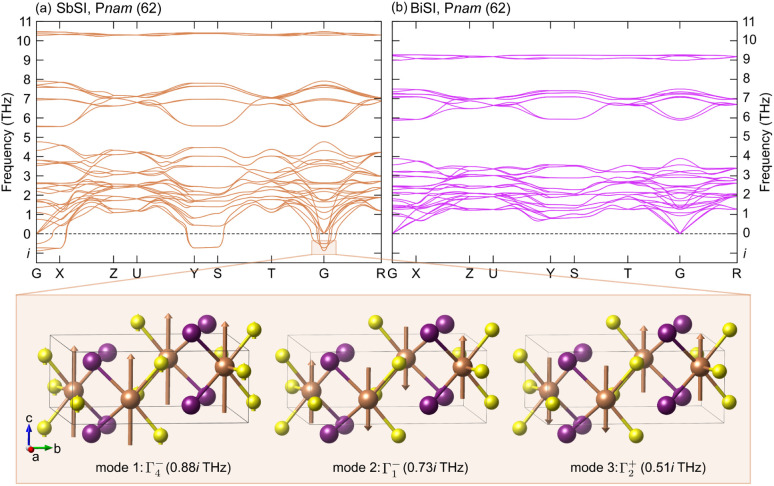
Computed phonon dispersions for (a) SbSI and (b) BiSI in the *Pnam* space group. The panels underneath the dispersion show the eigenvectors of the three unstable modes at the *Γ* point, with displacements shown relative to iodine (I).

Temperature dependent maps for *x* = 0 and *x* = 0.10 are shown in Fig. 4c and d. The softening of the low-frequency mode below 50 cm^−1^, associated with the Sb/Bi–S basal bonds (see again Fig. 3a and 5), follows the structural distortion responsible for the symmetry lowering at the ferroelectric transition.^34^ For *x* = 0, a clear softening is observed below RT (see dotted lines in Fig. 4c), followed by a non-zero SHG response, as shown in Fig. 4e. This reflects the lack of inversion symmetry below *T*_c_ (*i.e.*, in the ferroelectric state). We consider the steep increase of the SHG signal to mark the *T*_c_, but we notice a tail at high temperature, indicating some precursor state (see Fig. S11). We note that intermediate antiferroelectric domains have been proposed at the FE to PE transition in SbSI,^72,73^ that may deserve further consideration but are beyond the scope of this work. The obtained *T*_c_ (*x* = 0) ≈ 300 K for SbSI is in good agreement with dielectric measurements.^29,39^ For *x* = 0.10, a similar phonon softening is observed below *T*_c_ (*x* = 0.10) ≈ 150 K, in qualitative agreement with the onset of the negative thermal expansion observed by SXRD. For *x* = 0.20 a weak transition is observed below *T*_c_ (*x* = 0.20) ≈ 100 K, while no softening is observed for *x* = 0.30 (see Fig. S12). The strong depression of the critical temperature for the ferroelectric distortion as a function of *x*(Bi) in Sb_1−*x*_Bi_*x*_SI is confirmed by the SHG measurements as shown in Fig. 4e, with no ferroelectric behavior expected for bismuth contents above *x*(Bi) > 0.3. The *T*_c_ dependence on *x*(Bi) as determined by our combined Raman and SHG measurements is in qualitative agreement with the SXRD analysis (see Fig. 4f), and with earlier measurements of the dielectric properties of mixed Sb–Bi crystals grown from the melt.^39^ The observation of the ferroelectric distortion in the average crystal structure as derived from the SXRD and the lack of compositional segregation as deduced from our EDS maps and point analysis indicates the depression of the T_c_ to reflect a bulk effect driven by Bi substitution. The possibility of domains at the nanoscale as well as dynamic distortions not resolved by the present analysis remains to be evaluated, as observed in related systems.^74^ We note that the depression of the ferroelectric distortion with Bi incorporation correlates with the marked decrease in the bandgap at low substitution levels. As we discuss below, the different lone-pair expression in BiSI compared to SbSI may underlie the dramatic impact of substitution of Sb by Bi on the structural, electronic, and ferroic properties of SbSI. These results highlight that a compromise between an optimized bandgap and retaining ferroelectric properties has to be considered when designing hetero-structures based on mixed Sb_1−*x*_Bi_*x*_SI compositions.

### DFT analysis

In order to address the reason behind the lack of ferroelectric distortion in BiSI, we computed phonon dispersions for the two end members SbSI and BiSI. The centrosymmetric *Pnam* phase of SbSI is a saddle point on the potential-energy surface that separates two equivalent minima belonging to the ferroelectric polymorph *Pna*2_1_ (no. 33). Consequently, imaginary frequencies are expected at the *Γ* point in the *Pnam* phase. Our calculation exhibits three such zone-center instabilities (Fig. 5a): *Γ*^−^_4_ (0.88 i THz), *Γ*^−^_1_ (0.73 i THz) and *Γ*^+^_2_ (0.51 i THz). Group–subgroup analysis shows that the *Γ*^−^_4_ mode leads to condensation into the polar subgroup *Pna*2_1_, with eigenvectors consisting of synchronous cation–anion displacements along the crystallographic *c*-axis (Fig. 5 (left-panel)); this distortion breaks the inversion symmetry and acts as the primary ferroelectric order parameter. The other two modes drive nonpolar distortions (Fig. 5 (middle and right-panels)): *Γ*^−^_1_ leads to *P*2_1_2_1_2_1_ (no. 19, point group 222), whereas *Γ*^+^_2_ lowers the symmetry to *P*2_1_/*c* (no. 14, point group 2/*m*). Freezing the *Γ*^−^_4_ displacement and fully relaxing the structure eliminates the competing *Γ*^−^_1_ and *Γ*^+^_2_ instabilities. Thus, the *Γ*^−^_4_ soft mode dominates the energy landscape, yielding a dynamically stable polar *Pna*2_1_ ground state (Fig. S13). Unlike SbSI, the *Pnam* phase of BiSI is dynamically stable: its phonon spectrum contains only real frequencies (Fig. 5b), indicating that there is no driving force for a *Pna*2_1_-type polar transition. Ferroelectric phase transitions are driven by the presence of an anharmonic double-well potential in the crystal. Bi has an atomic radius approximately 0.16 Å larger than that of Sb,^75^ leading to tighter atomic packing and less space for off-center displacements along the *c*-axis.^76^ This results in a more harmonic potential and reduced anharmonicity in *Pnam* BiSI, thereby suppressing the ferroelectric phase transition.

Next, we examine the effect of Sb and Bi lone pairs in SbSI and BiSI to understand their potential role in inducing ferroelectricity. Lone-pair electrons can become stereochemically active by localizing asymmetrically (*i.e.*, tilting) around the cation, thereby breaking the inversion symmetry of the crystal. However, such off-centering requires sufficient spatial freedom within the lattice.

**Fig. 6:**
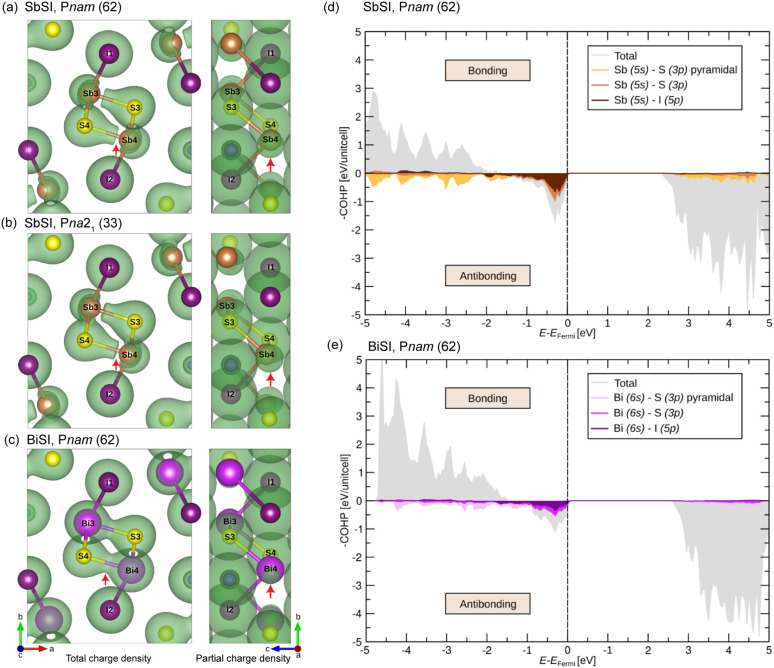
Total (left-panels) and partial (right-panels) charge–density maps for (a) SbSI, *Pnam*; (b) SbSI, polar *Pna*2_1_; and (c) BiSI, *Pnam*. The partial charge density is integrated over electronic states 0–2 eV below the Fermi energy (*E*_Fermi_). Red arrows in the left panels mark nodes (zero-density regions) and hybridized electron density, whereas those in the right panels highlight symmetric (centered) and asymmetric (tilted) lone-pair localization. Isosurfaces of the total and partial charge densities are visualized with isovalues of 0.05 and 0.015 e Å^−3^, respectively. Crystal-orbital Hamilton-population (COHP) analysis of the most relevant orbitals for the non-polar orthorhombic *Pnam* phase of (d) SbSI and (e) BiSI.

As shown by our partial charge density analysis, the lone pair of Sb in SbSI is symmetric (centered) around the Sb atom in the centrosymmetric *Pnam* phase (Fig. 6a, right panel). In contrast, in the polar *Pna*2_1_ phase, the lone pairs are asymmetric (tilted) toward the elongated Sb–I and Sb–S bonds (Fig. 6b, right panel). This observation suggests that the relatively compact Sb 5s orbital provides sufficient spatial flexibility for the lone pair to localize off-center, thereby promoting a polar distortion.

In contrast, BiSI exhibits significantly reduced lone-pair electron density on the Bi atom relative to Sb (Fig. 6c, right panel). The larger size of the Bi 6s orbital makes the lone pair more diffuse and delocalized, suppressing stereochemical activity. As a result, BiSI retains a centrosymmetric, non-polar *Pnam* crystal structure.

Finally, crystal orbital Hamilton population (COHP) analysis was conducted to examine the bonding characteristics, focusing on the antibonding states attributed to stereochemically active lone pairs. The Sb/Bi p orbitals contribute to the bonding states in our COHP analysis; however, we find no indication that they drive the ferroelectric transition; therefore, they are not shown in Fig. 6d and e. In the *Pnam* phase, SbSI exhibits sharp antibonding peaks near the Fermi energy, as the compact Sb 5s orbital hybridizes only weakly with the S 3p and I 5p orbitals (Fig. 6d). The pronounced localization of these states is corroborated by the presence of a node in the total charge density (Fig. 6a, left panel), which results from the weak involvement of the stereochemically active Sb-centered lone pair in antibonding interactions (Fig. 6a, right panel).

BiSI, on the other hand, exhibits broader antibonding states near the Fermi energy due to stronger hybridization between the Bi 6s orbital and the S 3p and I 5p orbitals, as compared to SbSI (Fig. 6e). Consequently, BiSI is stabilized by the less localized nature of these antibonding states near the Fermi level, in contrast to the more pronounced antibonding states observed in SbSI. This effect is evident in the enhanced orbital hybridization (Fig. 6c, left panel), indicating greater involvement of the Bi lone pair in antibonding interactions, which can be seen from the reduced electron density of the lone pair on the Bi atom (Fig. 6c, right panel).

In summary, our DFT results show ferroelectricity in SbSI but not in BiSI, which we associate with a lesser involvement of the more compact Sb lone pair in bonding and hence a greater stereochemical activity of the lone pairs in SbSI compared to BiSI.

## Conclusions

We have described the effect of Sb by Bi substitution on the structural, electronic, and ferroelectric properties across the Sb_1−*x*_Bi_*x*_SI solid solution by combining temperature-dependent synchrotron X-ray diffraction, UV-Vis spectroscopy, and combined Raman and second-harmonic generation measurements. We find that Bi substitution induces a pronounced anisotropic evolution of the unit cell, tunes the bandgap down to 1.5 eV, and progressively suppresses ferroelectricity, as evidenced by the loss of soft-phonon modes and second-harmonic generation. The lack of ferroelectric instability in BiSI has been confirmed by DFT calculations, which have also highlighted the key role of the lone pair expression in the ferroic behaviour. From our charge density and COHP analysis, the lone pair in Sb(Bi)SI is shown to not only impose structural constraint but to also play a central role in driving the polar distortion in the ferroelectric state. In particular, the increased hybridization *n*s^2^ (Sb, Bi)–*n*p (S, I) seems to be behind the suppression of ferroelectricity in Bi-rich compositions. The progressive tuning of the bandgap values in Sb_1−*x*_Bi_*x*_SI may allow band alignment in heterojunctions for improved solar absorption efficiency. Furthermore, the concomitant modulation of the ferroelectric distortion with Bi substitution provides additional perspectives for the design of optoelectronic devices with engineered ferroic and optical properties.

## Author contributions

FvR and SL designed the project. SL synthesized the samples. SL, AM, VM, and JT performed the measurements. HS and UA performed the DFT calculations. All authors contributed to the analysis and interpretation of the data. FvR, SL, HS, and UA wrote the manuscript with contributions from all authors.

## Conflicts of interest

There are no conflicts to declare.

## Supplementary Material

TA-014-D5TA07038D-s001

## Data Availability

The data supporting this article have been included as part of the supplementary information (SI). Supplementary information is available. See DOI: https://doi.org/10.1039/d5ta07038d.
