# Supplementary material for: Tailoring optical and ferroelectric properties in Sb1−xBixSI van der Waals chalcohalides towards solar absorber applications
Source: J Mater Chem A Mater. 2025 Nov 27;14(3):1681–90. doi: 10.1039/d5ta07038d (PMC12658473; doi:10.1039/d5ta07038d)
Supplement: TA-014-D5TA07038D-s001 [file TA-014-D5TA07038D-s001.pdf]

Supplementary Information (SI) for Journal of Materials Chemistry A.
This journal is © The Royal Society of Chemistry 2025

Supplementary Information

Tailoring Optical and Ferroelectric Properties in $Sb_{1-x}Bi_xSI$ van der Waals Chalcohalides towards Solar Absorber Applications

Sara A. López-Paz *[a], Harish K. Singh [b], Alba S. J. Mendez [c], Volodymyr Multian [d], Jeremie Teyssier [d], Ulrich Aschauer [b] and Fabian O. von Rohr*[d]

Table S1. Atomic and cell parameters obtained from the Rietveld refinement of the SXRD pattern for SbSI at 300 K with Pnam and $Pna2_1$ space groups.

Pnam						
Atom	*Site*	*x*	*y*	*z*	*Biso (Å2)*	*Occ.*
Sb	*4c*	0.1199(2)	0.1232(2)	0.25	1.91(5)	1
S	*4c*	0.8445(6)	0.0478(5)	0.25	1.4(1)	1
I	*4c*	0.5074(2)	0.8276(1)	0.25	1.73(5)	1
a	8.5326(4)					
b	10.1406(5)					
c	4.1042(2)					
V	355.13(3)					
RBragg	2.36	Rp	0.647	Rwp	1.02	
Pna21						
Atom	*Site*	*x*	*y*	*z*	*Biso (Å2)*	*Occ.*
Sb	*4a*	0.1195(1)	0.1233(1)	0.288(1)	1.1(1)	1
S	*4a*	0.8440(5)	0.0494(4)	0.281(4)	0.68(4)	1
I	*4a*	0.5075(2)	0.8279(1)	0.252(1)	1.15(7)	1
a	8.5327(3)					
b	10.1416(5)					
c	4.1045(2)					
V	355.19 (3)					
RBragg	2.11	Rp	0.594	Rwp	0.935	

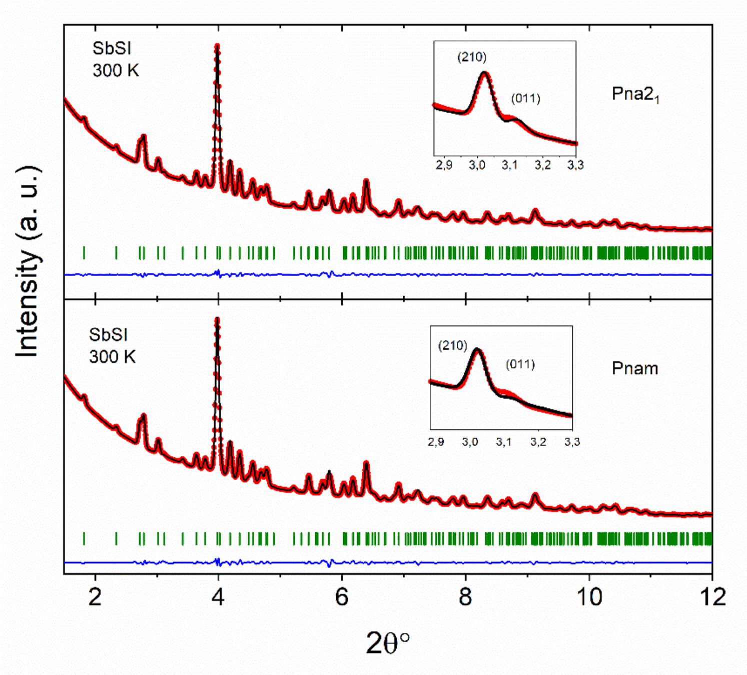

Figure S1. Refined SXRD pattern for SbSI at 300 K within the Pnam and $Pna2_1$ space groups.

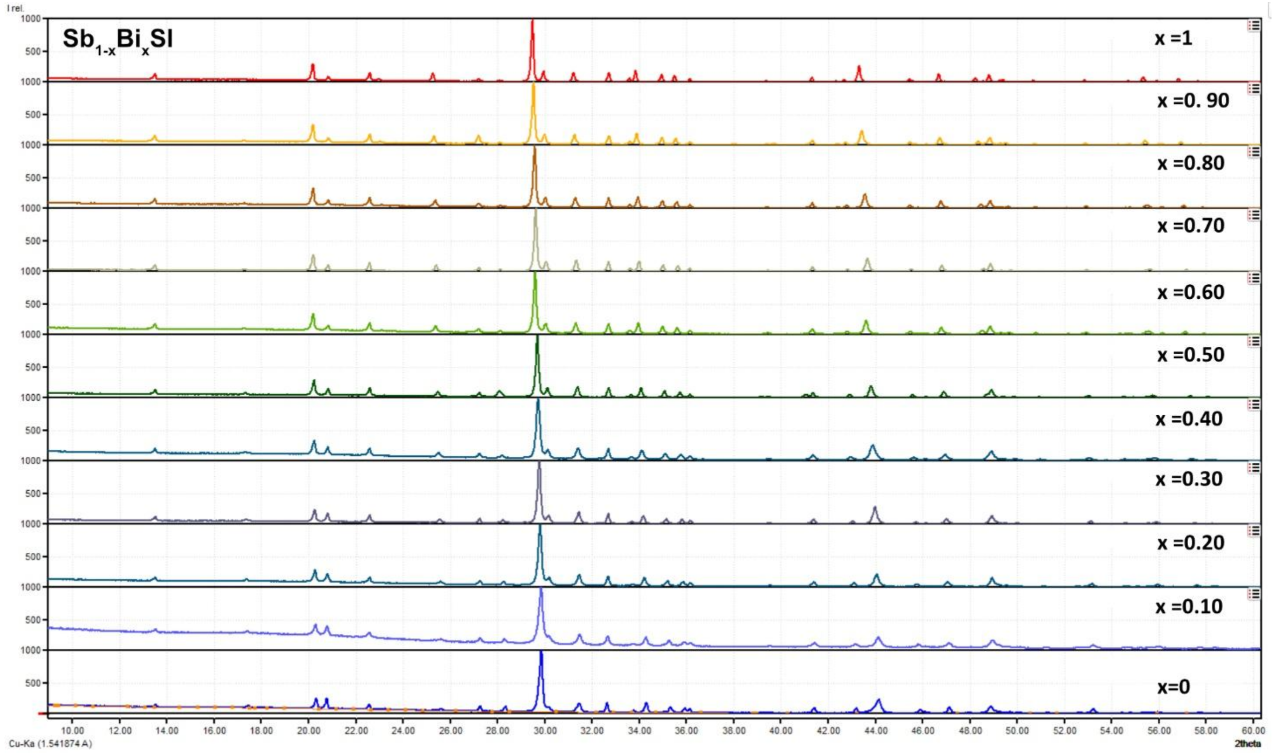

Figure S2. Room temperature PXRD patterns for the $Sb_{1-x}Bi_xSI$ (x = 0-1) solid solution

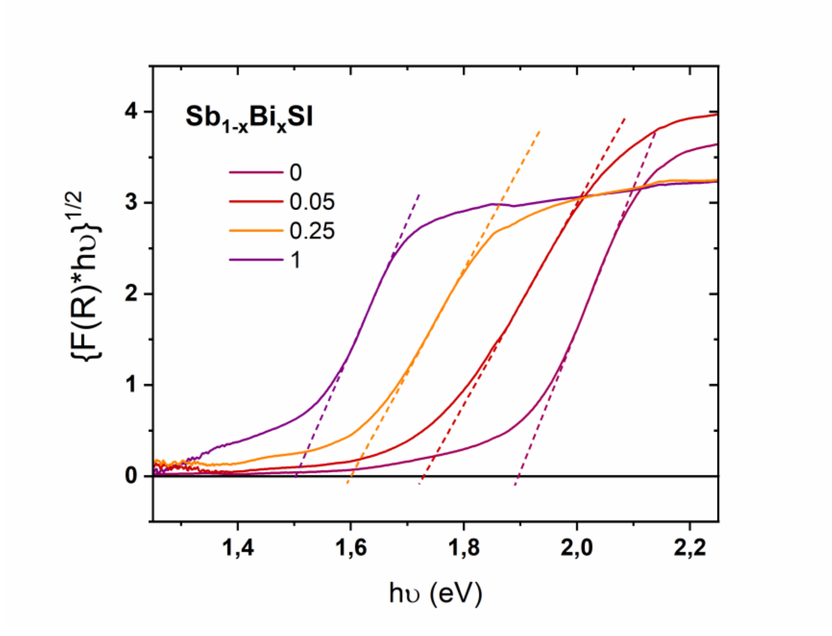

Figure S3. Tauc plots for selected $Sb_{1-x}Bi_xSI$ (x = 0, 0.05, 0.25 and 1) compositions. The bandgap is calculated from the extrapolation of the linear region.

Table S2. Atomic and cell parameters obtained from the Rietveld refinement of the SXRD pattern for SbSI at 100 K with Pnam and $Pna2_1$ space groups.

Pnam						
Atom	*Site*	*x*	*y*	*z*	*Biso (Å2)*	*Occ.*
Sb	*4c*	0.1208(2)	0.1219(2)	0.25	1.31(6)	1
S	*4c*	0.8461(8)	0.0445(6)	0.25	1.2(1)	1
I	*4c*	0.5071(2)	0.8278(1)	0.25	0.73(5)	1
a	8.4964(4)					
b	10.0790(6)					
c	4.1218(2)					
V	352.97(3)					
RBragg	4.15	Rp	0.921	Rwp	1.46	
Pna21						
Atom	*Site*	*x*	*y*	*z*	*Biso (Å2)*	*Occ.*
Sb	*4a*	0.1199(1)	0.1235(1)	0.3141(5)	0.39(4)	1
S	*4a*	0.8442(5)	0.0490(4)	0.274(2)	0.65(9)	1
I	*4a*	0.5075(2)	0.8279(1)	0.249 (2)	0.31(4)	1
a	8.4959(3)					
b	10.0763(4)					
c	4.1222(2)					
V	352.90(2)					
RBragg	1.98	Rp	0.596	Rwp	0.935	

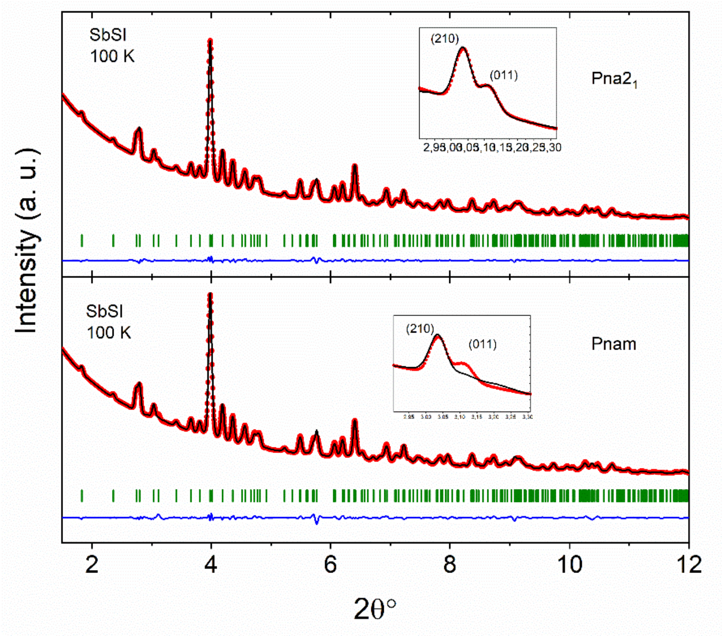

Figure S4. Refined SXRD pattern for SbSI at 100 K within the Pnam and $Pna2_1$ space groups.

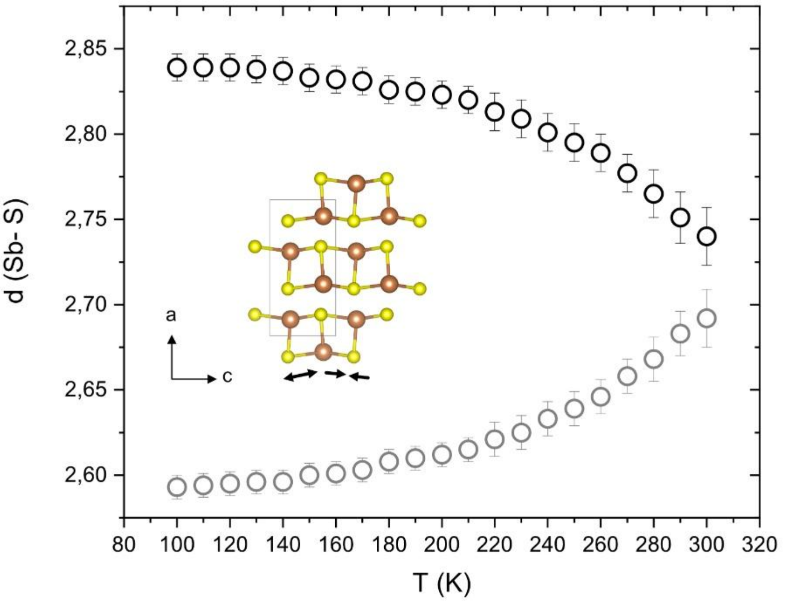

Figure S5. Temperature dependence of the Sb-S bond length in SbSI as derived from refinement of the SXRD patterns in the 100-300 K range, within the $Pna2_1$ space group.

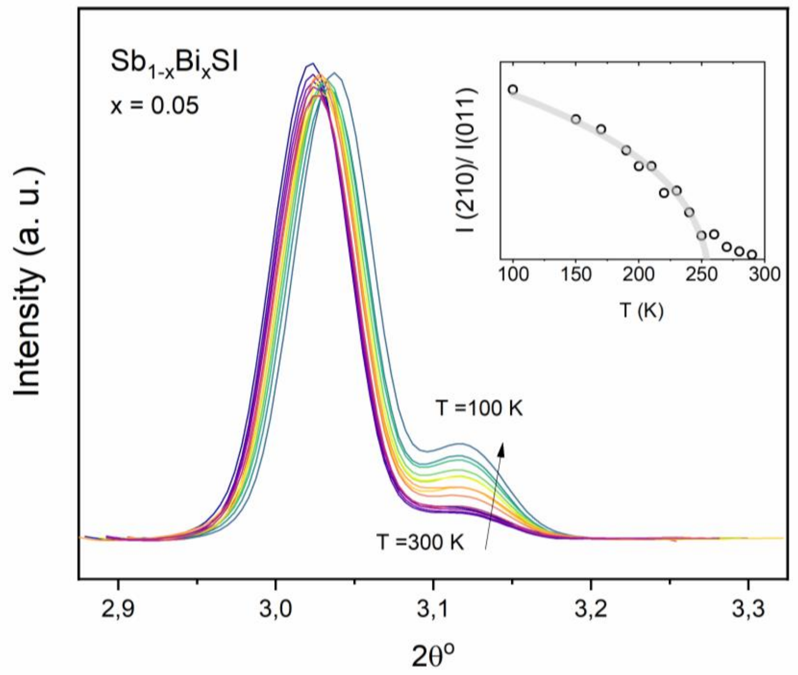

Figure S6. Temperature dependence of the intensity of the (210) and (011) reflections for $Sb_{1-x}Bi_xSI$ x=0.05.

Table S3. Cell parameters obtained from Rietveld refinement of the SXRD pattern for $Sb_{1-x}Bi_xSI$ at 300 K within the Pnam space group.

**x**	**a**	**b**	**c**	**V**
0	8.5328	10.1417	4.1045	355.19
0.05	8.5330	10.1626	4.1014	355.66
0.1	8.5300	10.1724	4.1026	355.98
0.15	8.5292	10.1786	4.1055	356.42
0.2	8.5276	10.1872	4.1101	357.05
0.3	8.5268	10.2045	4.1152	358.07
0.4	8.5253	10.2203	4.1242	359.34
0.5	8.5227	10.2324	4.1315	360.30
0.67	8.5200	10.2539	4.1509	362.63
0.72	8.5190	10.2549	4.1544	362.93
1	8.516 0	10.2639	4.1765	365.05

Table S4: Comparison of lattice parameters from DFT calculations (0 K) and experimental measurements. Experimental values correspond to 100 K for SbSI and 300 K for BiSI.

Difference (%) = ((DFT − Experimental) / Experimental) × 100

Compounds	Space group	Lattice axis	DFT (Å)	Experiment (Å)	Difference (%)
SbSI	Pnam (62)	a	8.5117	8.4964	+0.180
		b	10.0312	10.0790	−0.474
		c	4.0910	4.1218	−0.748
SbSI	$Pna2_1$ (33)	a	8.4945	8.4959	−0.016
		b	10.0022	10.0763	−0.735
		c	4.1672	4.1222	+1.092
BiSI	Pnam (62)	a	8.533	8.5160	+0.200
		b	10.081	10.2639	−1.785
		c	4.218	4.1765	+0.991

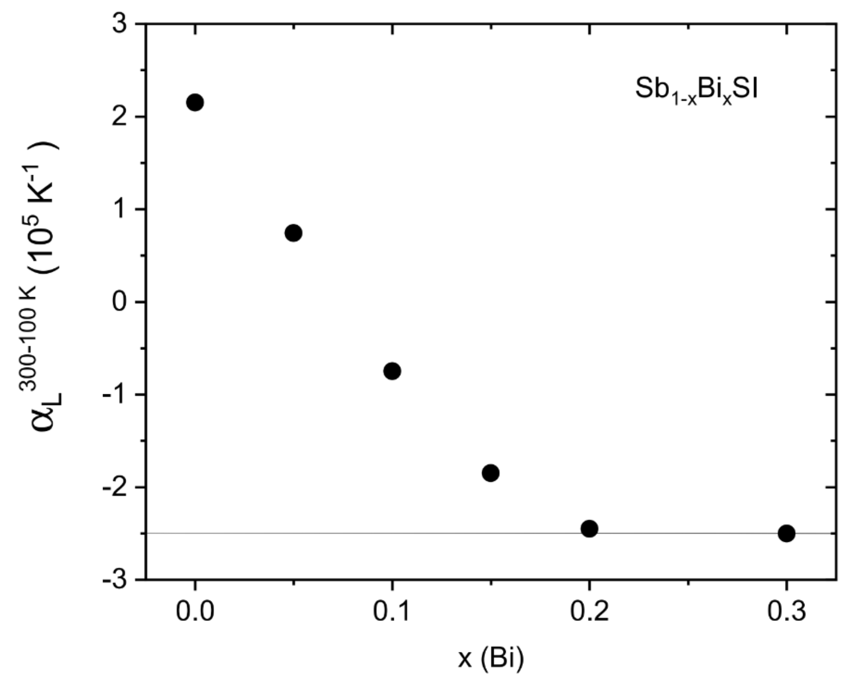

Figure S7. Thermal expansion coefficient α between 300 K and 100 K as for $Sb_{1-x}Bi_xSI$.

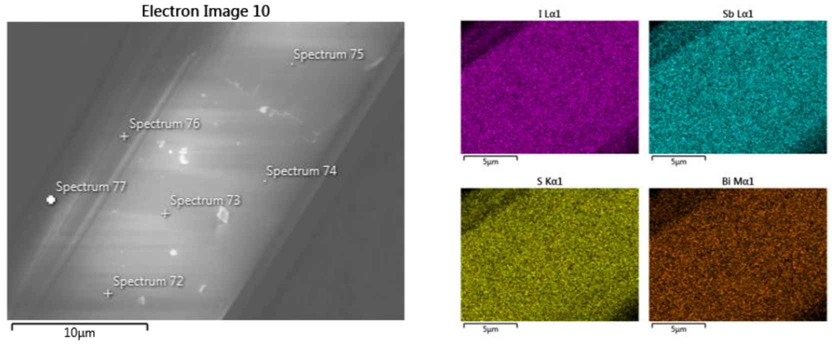

Spectrum Label	Spectrum 72	Spectrum 73	Spectrum 74	Spectrum 75	Spectrum 76	Spectrum 77
S	32.09	32.12	31.28	31.62	33.19	32.58
Sb	31.07	31.50	31.31	31.31	31.15	31.00
I	32.71	32.47	33.35	33.16	31.73	32.37
Bi	4.14	3.91	4.06	3.91	3.94	4.05
Total	100.00	100.00	100.00	100.00	100.00	100.00

Figure S8. SEM micrograph and corresponding EDS mapping for $Sb_{1-x}Bi_xSI$ (x =0.10).

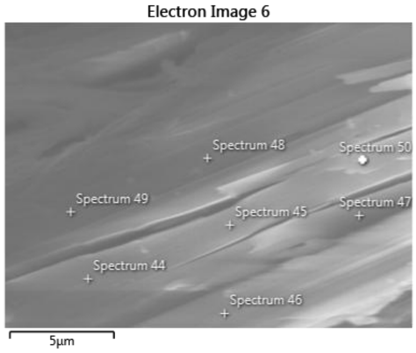

Spectrum Label	Spectrum 44	Spectrum 45	Spectrum 46	Spectrum 47	Spectrum 48	Spectrum 49	Spectrum 50
S	35.03	34.53	35.75	34.64	35.43	35.76	33.82
Sb	28.01	28.37	27.53	27.91	27.25	27.24	28.72
I	30.47	30.60	29.82	30.80	30.30	29.92	30.60
Bi	6.49	6.50	6.90	6.66	7.02	7.08	6.86
Total	100.00	100.00	100.00	100.00	100.00	100.00	100.00

Figure S9. SEM micrograph and corresponding EDS mapping for $Sb_{1-x}Bi_xSI$ (x =0.20).

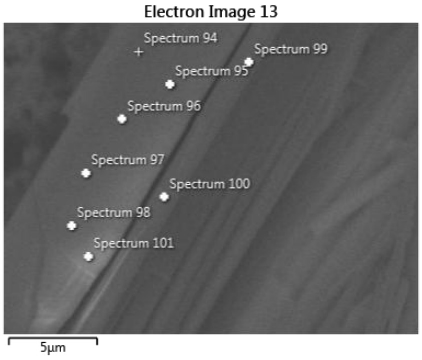

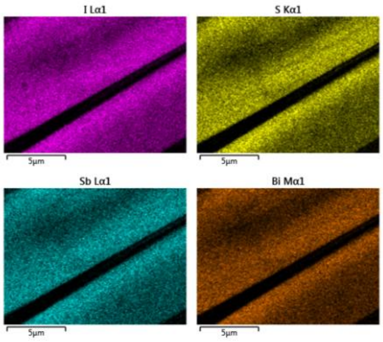

Spectrum Label	Spectrum 94	Spectrum 95	Spectrum 96	Spectrum 97	Spectrum 98	Spectrum 99	Spectrum 100	Spectrum 101
S	33.90	33.40	33.74	33.95	34.18	33.48	33.74	33.87
Sb	23.76	24.12	24.10	23.68	23.78	24.19	24.18	23.93
I	31.60	32.03	31.73	31.73	31.61	31.94	31.63	31.79
Bi	10.75	10.45	10.43	10.63	10.43	10.38	10.45	10.41
Total	100.00	100.00	100.00	100.00	100.00	100.00	100.00	100.00

Figure S10. SEM micrograph and corresponding EDS mapping for $Sb_{1-x}Bi_xSI$ (x =0.30).

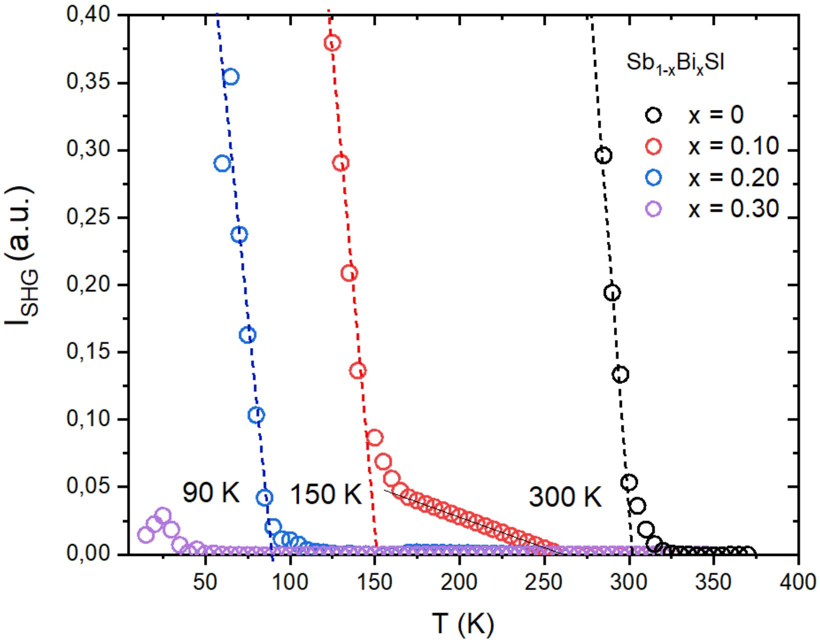

Figure S11. SHG measurements for $Sb_{1-x}Bi_xSI$ (x =0.-0.30). The Tc is determined by extrapolation of the step increase on the SHG intensity.

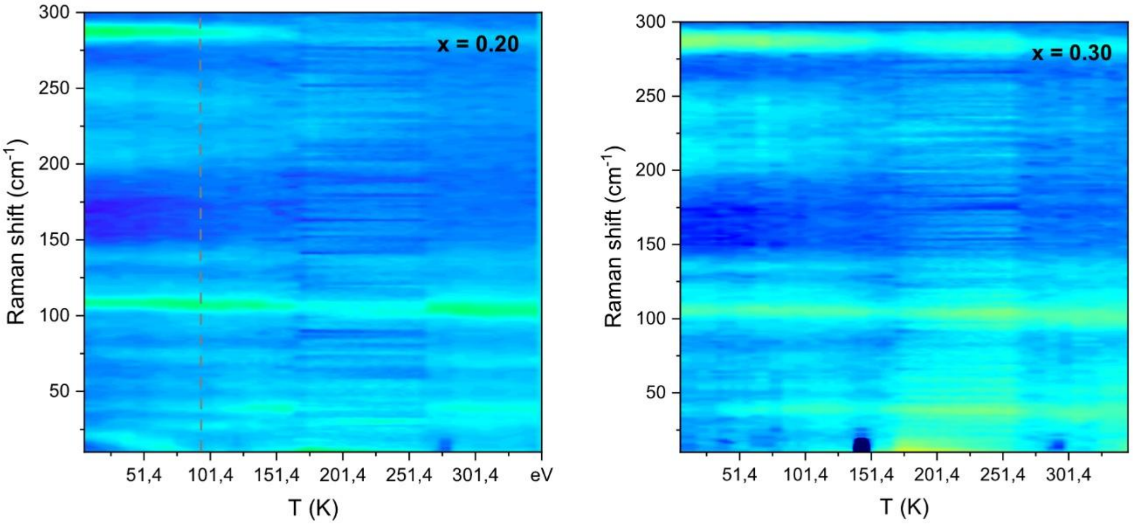

Figure S12. Temperature dependent Raman color plots for $Sb_{1-x}Bi_xSI$ (x =0.20 and x=0.3).

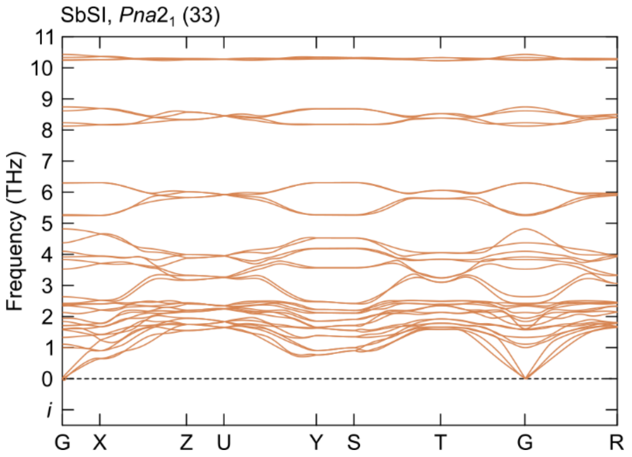

Figure S13. Computed phonon dispersions for SbSI in Pna2$_1$ (33) space group.

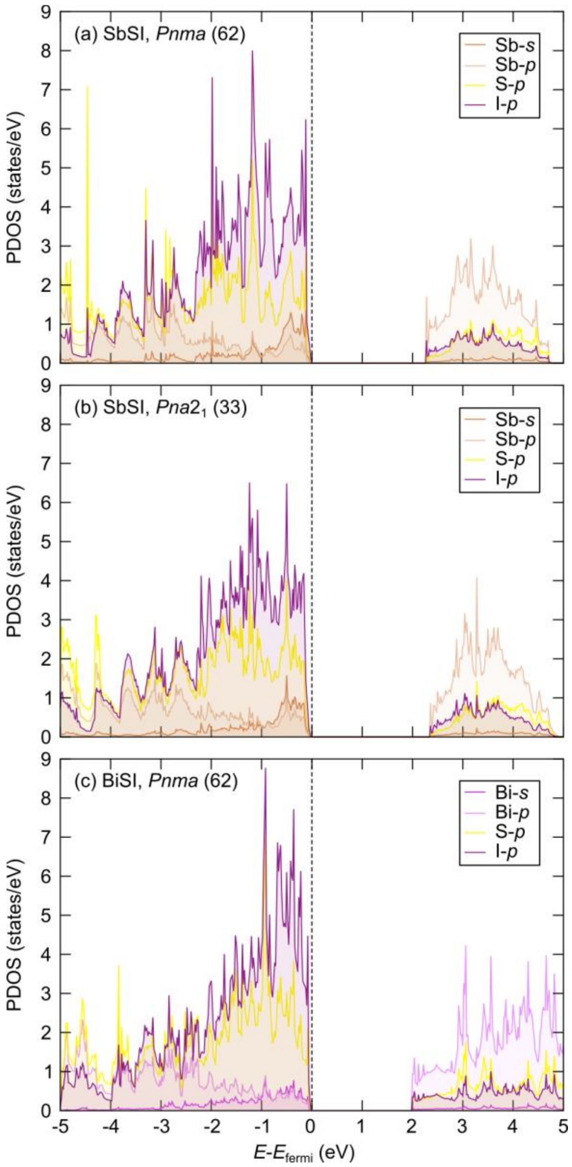

Figure S14. Projected densities of states including spin-orbit coupling for (a) SbSI in the Pnma space group, (b) SbSI in the Pna2$_1$ space group, and (c) BiSI in the Pnma space group.

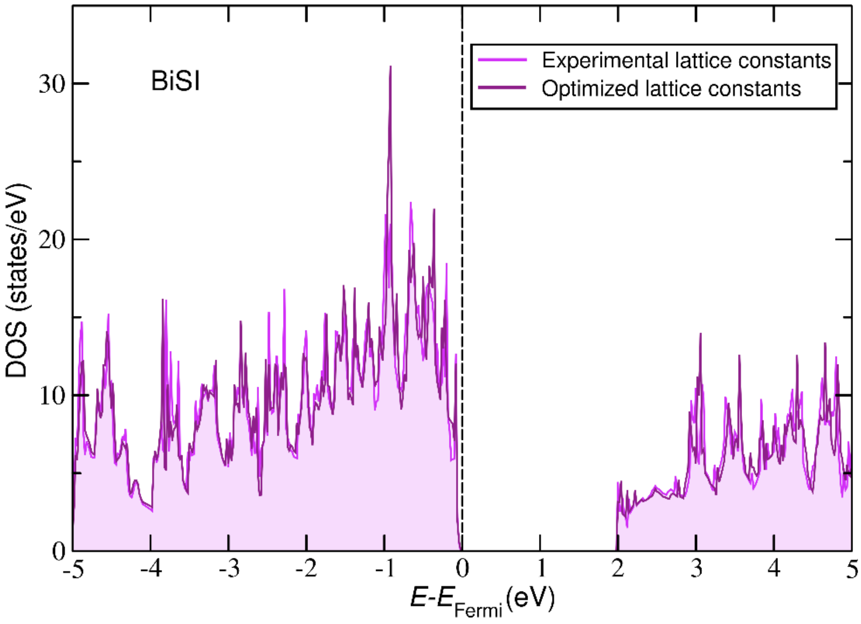

Figure S15: The calculated DOS using spin-orbit coupling (SOC) for BiSI using the experimental and fully optimized lattice constants.

Supp Note 1. DRS analysis

Diffuse reflectance spectra can be transformed into absorption spectra through the Kubelka–Munk function (F(R∞). eq 1) (32)

$F(R\infty)=KS=(1-R\infty)22R\infty$

(1)

where R∞ is the absolute reflectance and K and S are the absorption and scattering coefficients. respectively. F(R∞) is proportional to the extinction coefficient (α). which can be expressed by eq 2. according to the Tauc method (33)

$(\alpha h\nu)1/\gamma=A(h\nu-E\text{g})$

(2)

where h is the Plank constant. ν is the photon frequency. Eg is the band-gap energy. and A is a constant. γ = 2 has been considered assuming an allowed indirect transition. Replacing α with F(R∞) results in the following expression (eq 3)

$F(R\infty)(h\nu)1/\gamma=A(h\nu-E\text{g})$

(3)

The band gap can thus be extracted from diffuse reflectance spectra transformed according to eq 3. Linear fitting of the Tauc plot followed by extrapolating to the x-axis intersection gives the band-gap value.
